# Delays without Mistakes: Response Time and Error Distributions in Dual-Task

**DOI:** 10.1371/journal.pone.0003196

**Published:** 2008-09-12

**Authors:** Juan Esteban Kamienkowski, Mariano Sigman

**Affiliations:** Integrative Neuroscience Laboratory, Physics Department, Facultad de Ciencias Exactas y Naturales, Universidad de Buenos Aires, Buenos Aires, Argentina; University of Minnesota, United States of America

## Abstract

**Background:**

When two tasks are presented within a short interval, a delay in the execution of the second task has been systematically observed. Psychological theorizing has argued that while sensory and motor operations can proceed in parallel, the coordination between these modules establishes a processing bottleneck. This model predicts that the timing but not the characteristics (duration, precision, variability…) of each processing stage are affected by interference. Thus, a critical test to this hypothesis is to explore whether the qualitiy of the decision is unaffected by a concurrent task.

**Methodology/Principal Findings:**

In number comparison–as in most decision comparison tasks with a scalar measure of the evidence–the extent to which two stimuli can be discriminated is determined by their ratio, referred as the Weber fraction. We investigated performance in a rapid succession of two non-symbolic comparison tasks (number comparison and tone discrimination) in which error rates in both tasks could be manipulated parametrically from chance to almost perfect. We observed that dual-task interference has a massive effect on RT but does not affect the error rates, or the distribution of errors as a function of the evidence.

**Conclusions/Significance:**

Our results imply that while the decision process itself is delayed during multiple task execution, its workings are unaffected by task interference, providing strong evidence in favor of a sequential model of task execution.

## Introduction

Interference experiments constitute a very powerful and experimental technique to understand the internal organization and architecture of a cognitive task. The logic of these experiments–which have been extensively explored in psychological research–resembles the classic scattering methodology in physics whereas the internal structure of an element (particle, molecule…) is understood by colliding it with an experimental probe. When two cognitive tasks are presented and executed within a short interval different manifestations of interference have been observed, even when they involve distinct sensory and motor modalities.

A classic demonstration of such interference effect is the Psychological Refractory Period: when the two tasks are speeded (subjects have to respond to two items as fast as possible) a systematic delay is observed in the execution of the second task [1]–[4]. Based on numerous experiments which manipulate the stimulus onset asynchrony (SOA), the task order, factors affecting either or both tasks, etc…, it has been concluded that while sensory and motor operations can proceed in parallel, the coordination between these modules establishes a serial processing bottleneck [2], [5]–[11]. The most convincing evidence in psychological experimentation in favor of this model comes from “slack” experiments [2], [7] in which the durations of specific stages are manipulated at different SOA values. One aspect of the logic of these experiments is simple and serves to illustrate the main ideas: manipulating the duration of a processing stage prior to the bottleneck should not affect response time (it does not help much to speed-up and arrive fast if there is a cue at the end of the path…). This observation has indeed been observed in numerous experimental setups. Evidence in favor of such scheduling and queuing of mental processes in dual task phenomenon comes from investigations of the cerebral basis of processing bottlenecks with Event Related Potential studies (ERPs). These studies have shown the bracketing of components with timing characteristics unaffected by dual-task (reflecting parallel processing)–although often modulated in amplitude by the concurrent task–and those manifesting a sequential, bottleneck delay [12]–[20].

An important aspect of this model is that the timing–but not the characteristics (duration, precision, variability…) of each processing stage are affected by interference. Beyond purely chronometric measures, this hypothesis establishes a critical prediction for simple decision tasks; If the processing stages involved in a cognitive task are merely rescheduled during dual task performance, the quality of the decision should be unaffected by a concurrent task. Here we set out to test this hypothesis in a very quantitative manner by exploring the distribution of errors in a dual-task procedure involving a non-symbolic decision task.

In number comparison–as in most decision one-dimensional comparison tasks–the extent to which two stimuli can be discriminated is determined by their ratio, referred as the Weber fraction [21]–[25], which establishes a measure of the resolution of the decision making process. The decision-making process has been modeled as a noisy integrator that accumulates evidence provided by the sensory system [26]–[34]. These models have been often used indistinctively in symbolic and non-symbolic tasks, although the emergence of symbols presents important qualitative and quantitative differences [24], [35]–[38]. For instance, it has been shown that mapping of quantity into a continuous line switches from a logarithmic to a linear scale with the emergence of symbols. This has been shown in developmental [39] as well as in cross-cultural studies [40]. These differences have been explained by theoretical models, which predict the existence of neurons entrained to numerosity information alone or paired to symbolic information. These neurons develop skewed receptive fields whose dispersion increases with numerosity (which become Gaussian and with fixed dispersions in a logarithmic scale) for numerosity alone. The very same neurons may show sharply tuned receptive fields which are Gaussian like in linear scale and with a constant dispersion for different numerosities when stimulated with symbolic quantities [41]–[43]. Since the implicit assumption of diffusion models is that evidence to reach a decision is conveyed by sensory neurons, these differences may become very important when modeling symbolic or non-symbolic decision. In part, the success of this modeling strategy in both forms of decisions may be explained by the fact that symbolic operation cannot completely bypass a highly automatic (and probably the default) circuit of non-symbolic operation [40], [44]–[46]. Yet, a fundamental difference which may pose an important challenge to formal accumulation models is the relation between error rates and response times. In previous studies in which the distribution of response times in a dual-task experiment were studied using accumulation models [11], [47], error rates were too low to be modeled and thus, the covariation between error rates and mean and dispersion of response times, an important prediction of the accumulation models [29], [37], [48]–[51] could not be tested [52]. The main aim of the present study was to explore these relations and how they are affected by interference with a concurrent task.

We investigated performance in a rapid succession of two non-symbolic comparison tasks (number comparison and tone discrimination) in which error rates in both tasks could be manipulated parametrically from chance to almost perfect. Consistent with the sequential model, we observed that dual-task interference has a massive effect on RT but does not affect the error rates, or the distribution of errors as a function of the evidence.

## Results and Discussion

### Task selection and execution in an analog dual-decision task

**Figure 1:**
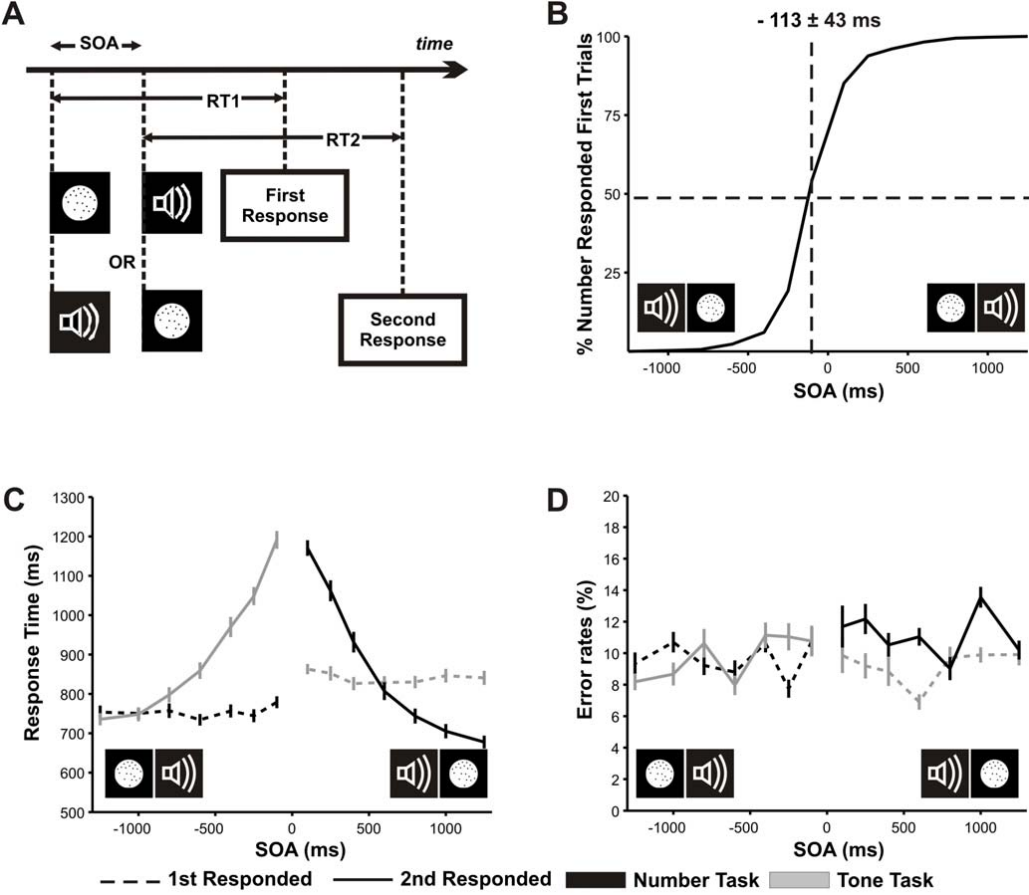
Task design and the effect of SOA on response order, RTs and errors. A) Experimental design. A number and a tone discrimination task were presented at a variable SOA. Task order was unknown and response times to each task were considered from its corresponding stimulus presentation. B) Response order as function of SOA. C,D) Response Time (RT) and Error rates as function of SOA. Positive SOA corresponds to number presented second trials. The classic PRP can be observed (C). RT2 (solid lines) decreases with a slope close to −1 for short SOA until it reaches a plateau. RT1 is unaffected by SOA (dashed lines). Error rates are unaffected by SOA (D). Both for Number and Tone task (black and gray lines respectively).

Participants were asked to respond as fast as possible to an auditory and a visual stimulus (Figure 1A). The auditory stimulus was a pure tone lasting 200 ms with a frequency chosen randomly from the following list: {350, 441, 556, 882, 1111, 1400} Hz Participants responded with the middle and annular finger of the left hand, whether the tone was higher or lower frequency than a fix reference set at 700 Hz. The visual stimulus was a set of dots in a circular display. The critical variable was the number of dots while intrinsic and extrinsic parameters (respectively density and occupied area of the set of dots) were equalized during the length of the experiment [22], [53], [54]. Participant responded using the right hand to indicate whether there were more or less dots than a fix reference, set at *20*. The number of dots varied from 10 to 40, sampling the number line uniformly in a log scale. The stimulus order was unpredicted for participants. Stimulus onset asynchrony (SOA) between both stimuli varied between 100 and 1250 ms.

Participants had the choice of determining which stimulus they would respond first. Note that although correlated, the order of presentation and of response is not necessarily identical for each trial. The dependence of task choice on SOA (Figure 1B), which follows a sigmoidal relation, indicates that selecting which task to respond first is determined, within a certain temporal jitter, by presentation order. At SOA = 0 (simultaneous presentation), “number responded first trials” corresponded to 74±2% of the total. The SOA value giving an unbalanced choice of 50% responses to each task–referred as 50% SOA–averaged across subjects, was 113±11 ms. The temporal interval from an SOA of 80% of “number responded to first trials” to a SOA of 80% of “tone responded to first trials” is 303±21 ms. These results are in very tight resemblance with a previous dual task study in which numbers were displayed in arabic digits or spelled words and subjects compared only between two tones [47].

### Effects of SOA on Response Times (RTs) and Error rates

An analysis of the dependence of RTs with SOA (Figure 1C) revealed a classic PRP effect. RTs to the first task (RT1) were mostly unaffected by SOA while response times to the second task (RT2) decreased with SOA. For “tone responded first trials” the slope of the regression is not significantly different than −1–as quantitatively predicted by the sequential bottleneck model–for *short SOAs*, henceforth used to refer to SOA values of {100, 250, 400} ms (mean value of the slope of the regressions for each individual subject: −0.80±0.10, t-test comparing the mean with −1: t = 2,07, p = 0.06, CI: [−1.01,−0.59]). For “number responded first” trials, the slope of the regression was negative but significantly larger than −1 (mean = −0.74±0.06, t = 4,53, p<0.001, CI: [−086,−0.61] ). For long RT2, mean values reached a plateau in which the responses become independent of SOA. While this is a classic observation in many PRP studies, we had previously found that in situations in which task order is unknown, RT1 increases for short SOAs. Here we did not replicate this result, probably because subjects had extensive training prior to the experiment. (t-test comparing RT1 at SOA = {100, 250} ms with SOA = {1000, 1250} ms for Number Task: t = 0,58, p = 0.57, CI: [−27.82,−48.51]; and for Tone Task: t = 0,41, p = 0.68, CI: [−29.85,−22.20]).

We then studied the dependence of the total number of errors (collapsed across all distances) as a function of SOA (Figure 1D). We did not see any significant change in the error rate with SOA either in the first or second responded task, regardless of whether the responded task was the number or the sound task (the four t-tests comparing mean error values between short and large SOAs for Number Responded first, Number Responded Second, Sound Responded First, Sound Responded Second had a p value larger than 0.1). Thus, the first and most important result of this paper is that, in striking contrast with the dependence of RTs–which shows a very significant increase is observed in RT2 as SOA shortens, error rates are completely unaffected by dual-task interference.

### Effects of Distance, Task Order and SOA on Error Rates

While the mean error rate is an informative estimator of the underlying processes of a cognitive task, more quantitative aspects and insights of the workings of the decision process can be understood by measuring the dependence of errors with the critical variable involved in the decision. In our experiment, the evidence is determined by the *Log numerical distance* and the precise probability of generating a response given a stimulus can be estimated using bayesian models based on maximum likelihood hypothesis [55], [56]. In the case of a number comparison task, under the assumption of a Log-Gaussian representation of numerosity [25], the probability that, for a given stimulus numerosity (*n*) and a fixed reference (*n_ref_*), the response time is larger (or smaller) can be calculated analytically (*P_larger_*) [21] and is determined by the following equation:
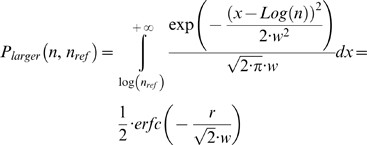
(1)where: *r* is *Log distance* (*r = Log(n/n_ref_)*), and *w* is the *Internal Weber Fraction* (see Appendix S1 for a derivation of this equation). This theoretical distribution was used to estimate the *internal weber fraction* (*w*) for different experimental conditions. In essence, the Weber fraction determines the resolution of the decision process.

**Figure 2:**
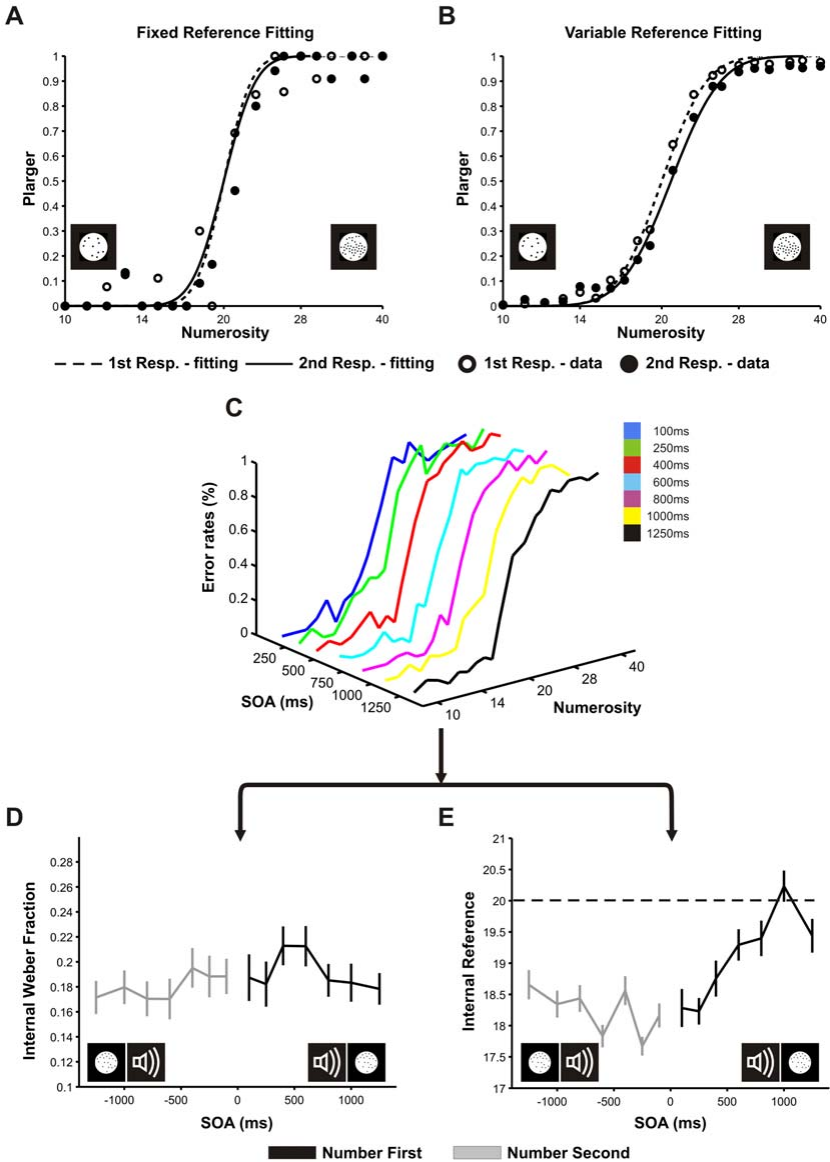
Interactions between interference (SOA) and decisional (log numerical distance) variables. A) Probability of a response “larger” as a function of the numerosity when the number task was responded first (open circles) and second (solid circles). Both curves can be fit by a sigmoidal function (dashed and solid lines respectively). The width of this function, which is an estimate of the internal Weber Fraction (*w*), is unaffected by task order. B) Same data as in A, fit to a sigmoid function with a varying reference. The internal reference (50% response) is closer to the objective reference (*20*) when the number task is responded first. C) Dependence of error rates as function of numerosity for different SOA values, represented in different colours. For number task responded second trials. Error rates and their distribution are unaffected by SOA. (SOA color labels: blue = 100 ms, green = 250 ms, red = 400 ms, cyan = 600 ms, magenta = 800 ms, yellow = 1000 ms and black = 1250 ms) D, E) The internal weber fraction is not affected by SOA (D), while the internal reference increase for larger SOAs when number task was presented second (E). Both parameters estimated with the same fit (equation 2). Black lines correspond to Number responded second trials and grey lines corresponds to Number responded first trials.

We measured *P_larger_* as a function of numerosity (Figure 2A) for n*umber responded first trials* and for *number responded second trials*, collapsing across all SOA values and fitted these experimentally obtained distributions to the theoretical prediction given by equation 1. Overall, all fits were accurate, as seen in figure 2A and as indicated by the small values of root mean square error (*RMSE),* indicating that the theoretical model provides an adequate description of our experimental data for all conditions (*RMSE*: number responded first trials 0.026 and number responded second trials 0.055). The *internal weber fraction* is not affected by task order (t-test comparing number responded first trials and number responded second trials: *w*: 0.16±0.01 and 0.18±0.02, t = 1.22, p = 0.24, CI: [−0.01,0.05]). The values we obtained of *w* are very close to other values reported previously in the literature [22]–[25].

In the previous fit we assumed that the internal reference–i.e. the numerical distance at which participants respond at chance–was set to the objective reference (fixed to twenty throughout the experiment). However, many number comparison studies have shown that there is a systematic sub-estimation of numerosity for large numbers [57]–[59], indicating that the internal and objective reference may not coincide. We thus performed a second fit of the data in which the internal Weber fraction and the internal reference were considered as free parameters (Figure 2B), fitting the data to the following equation:
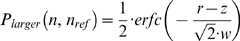
(2)Where *r*, and *w* are the same than in equation 1 and *z* is the log distance between the internal and the objective reference. The fits improved slightly for number responded second trial after inclusion of the internal reference (*RMSE*: number responded first trials 0.027 and number responded second trials 0.036). The internal Weber fraction, *w* is not affected by task response order (t-test comparing number responded first trials and number responded second trials: *w*: 0.15±0.01 and 0.17±0.01, t = 1.12, p = 0.28, CI: [−0.01, 0.04]). The obtained values of *w* are not significantly different from the values we had obtained in the previous fit. Interestingly, we observed a significant change in the internal reference as a function of task order, (paired t-test: t = 2.54, p<0.05, CI: [0.00, 0.07]). Thus, the internal reference seems to be the sole parameter in the decision making process which is affected by task order–a broad measure of interference of a concurrent task.

Having found that error rates are insensitive to SOA and that the Weber fraction is independent of task order, we explored whether the Weber fraction changes with SOA. We focus this analysis on the numerical task, because in our experiment numerical distances are sampled at a higher resolution than frequency distances and also because the internal numeric reference proved to be more stable than the auditory reference, which showed some drifts during the experiment. Similar results were found however when this analysis is performed in the responses to the auditory task (reported in Table 1). We first analyzed the functional dependence of error rates with distance, for different values of SOA (Figure 2C) To provide a quantitative comparison for different SOA values, we estimated the internal Weber fraction (*w*) and internal reference using equation 2, for each individual subject and each value of SOA in the number responded second trials. We submitted this data to an ANOVA and, in close correspondence with the task order manipulation, we did not see any effect of SOA on the Weber fraction (df = 6, F = 0.08, p>0.9) indicating that the tuning curves for errors is not affected by interference (Figure 2D). The SOA resulted, however, on a monotonic change in the reference as SOA increased, when the number task was performed second (Figure 2E). Previous studies on numerosity estimation have reported a tendency to underestimate the actual value [57], [59], although this tendency can be reverted with proper calibration [60]. In this study, we calibrated subjects, presenting them the reference (in the same visual display as the stimuli used in the experiment) before the beginning of each experimental block and, indeed, we observed that the subjective reference is very close to the objective reference. The unexpected finding of an increase in the subjective reference when the number task is presented second and the monotonic increase with SOA suggest a small departure from the purely passive sequential model and may involve a role of attention in the perception of numerosity which would be interesting to explore and study in detail in further investigations.

**Table 1:** Effects of SOA and distance in response times and errors of both tasks.

		Number task responded first trials			Number task responded second trials		
Variable	Parameter	(df)	F	P	(df)	F	P
*RT (Number task)*	*SOA*	2	0,05	p>0.05	**2**	**15,26**	**p<0.001**
*RT (Number task)*	*DIST*	**1**	**4,66**	**p<0.05**	**1**	**11,49**	**p<0.01**
*RT (Number task)*	SOA*DIST	2	0,00	p>0.05	2	0,06	p>0.05
*Errors rate (Number task)*	*SOA*	2	0,63	p>0.05	2	0,60	p>0.05
*Errors rate (Number task)*	*DIST*	**1**	**150,93**	**p<0.0001**	**1**	**116,57**	**p<0.0001**
*Errors rate (Number task)*	SOA*DIST	2	2,59	p>0.05	2	2,80	p>0.05
*RT (Tone task)*	*SOA*	2	0,04	p>0.05	**2**	**16,44**	**p<0.001**
*RT (Tone task)*	*DIST*	2	3,15	p>0.05	2	2,80	p>0.05
*RT (Tone task)*	SOA*DIST	4	1,47	p>0.05	4	0,98	p>0.05
*Errors rate (Tone task)*	*SOA*	2	0,22	p>0.05	2	1,27	p>0.05
*Errors rate (Tone task)*	*DIST*	**2**	**77,87**	**p<0.0001**	**2**	**36,89**	**p<0.0001**
*Errors rate (Tone task)*	SOA*DIST	4	1,11	p>0.05	4	0,24	p>0.05

In summary, we studied the functional dependencies of response times and error rates in a dual-task experiment where each task involved a non-symbolic decision. For response times we replicated the main features observed in prior dual-task studies, showing a strong delay in the execution of the second task which decreases as SOA increases and no effect on the first responded task. On the contrary, we did not observe any significant effect on the total number of errors or on the distribution of errors as a function of the numerical distance between the target and the reference, suggesting that the decision process itself is delayed but its workings are unaffected by task interference.

## Methods

### Participants

A total of 16 participants were involved in this study (six males, age 25±4). Participants were all native Spanish speakers. All subjects gave written informed consent and were naive about the aims of the experiment. All the experiments described in this paper were reviews and approved by the ethicss comittee: “Comité de Ética del Centro de Educación Médica e Investigaciones Clínicas “Norberto Quirno” (CEMIC)” qualified by the Department of Health and Human Services (HHS, USA): IRb00001745 - IORG 0001315.

### Stimuli and Tasks

#### Number Task

The visual stimulus consisted of a white circle containing a variable set of black dots and was shown in the center of the screen for 200 ms. The number of dots (*n*) varied between 10 and 40, equidistant in a log scale, and centered in the objective reference (*20*) which was unchanged during the experiment. The spatial distribution of the dots was varied pseudo-randomly, equating the extrinsic and intrinsic properties of the stimuli (i.e. total luminance and item size) similar to the stimuli used in previous number comparison studies [22], [23], [53], [54], [61].

Participants responded to the number task with a single key press using middle and index fingers of the right hand; to indicate whether the number of dots was larger or smaller than a fixed reference (*n_ref_ = 20*). Stimuli were shown in a 14-in monitor with a refresh rate of 60 Hz. Participants sat 60 cm from the screen.

#### Tone Task

The auditory stimulus was a pure tone mixed with a 20% of white noise (to avoid highly picked spectral content) and was presented for 200 ms. The frequency of the tone varied between 350 Hz and 1400 Hz, equidistant in a log scale. Three tones had frequencies under 700 Hz and three above 700 Hz. We determined the scale and the number of tones empirically to equate the difficulty of both tasks (Figure 1C and 1D). Participants responded to this task with a single key press using middle and index fingers of the left hand; indicating whether the tone was higher or lower than a fixed reference (*700 Hz*). Auditory stimulation was provided through headphones.

### General Procedure

Participants were asked to perform two tasks, with the clear instruction that they had to respond accurately and as fast as possible to each one as its corresponding stimulus was delivered. We emphasized that both tasks were equally important and that proper completion of a trial involved rapid and correct performance in both tasks.

The experiment was divided in blocks of 42 trials. In each block, the delay in the Stimulus Onset Asynchrony (SOA) and the order of the two tasks changed pseudo randomly from trial to trial, sampling the SOA values {100, 250, 400, 600, 800, 1000, 1250} ms and stimulus presentation orders {Number First, Tone First},. Thus, there were a total of 14 trial types which were repeated three times within one block (see Figure 1A). The temporal interval between the end of second stimuli of one trial and the beginning of next trial (i.e. the time to respond to the second task) varied randomly between 2300 and 2600 ms.

Prior to the beginning of the experiment, participants were shown the numerical and auditory references (10 repetitions). To avoid major drifts of the internal reference, we also presented the auditory and numerical references (2 repetitions) in the beginning of each block. Participants performed a total of 17 blocks (714 trials).

Before data collection, participants were trained for one to three blocks. Response performance was monitored online and participants did not proceed to the experiment until proper performance was assured (i.e. they did not grouped both responses and response times were bellow 1300 ms for 10 consecutive trials).

### Data analysis

#### Definitions

All the analyses described here for Response Times (RTs) were done only on correct responses. Trials in which the response times to one task exceeded 2.5 times mean RT and trials in which participants responded to only one task were excluded (less than 10% of the trials). All the statistics were done using the MATLAB, and in all ANOVAs, participants were treated as a random factor. Unless otherwise noted, all the t-tests were paired t-tests for values calculated for each individual subject (N = 16 in all cases).

The **numerical distance** is defined as the absolute value of the difference between the presented number and the reference (fixed to 20 throughout the experiment). Since for the most part dependencies with distance are logarithmic, we refer to **log numerical distance** (***r***) between the presented numerosity (***n***) and the fixed reference (***n_ref_***) as
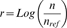
(3)


For certain analysis we categorized the SOA values in “**short SOA**” which refers to SOA values of {100, 250, 400} ms and “**large SOA**” as SOA = {600, 800, 1000, 1250} (see Figure 1C). Similarly, the log distance was categorized in **close** (*r*<0.25) and **far** (*r*>0.25, see Figure 2A and 2B).

#### Experimental variables estimated through regressions

To quantitatively measure response choice preference, response order was adjusted with a shifted sigmoid function, with two free parameters, *a* and *b*:
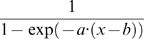
(4)


The parameter *b* corresponds to the SOA at which both tasks were responded first with equal probability (**50%SOA**, Figure 1B). We first estimated the 50% SOA on a subject by subject basis (by performing a fit for each individual subject) and then averaged and submitted to statistical analysis. All the fits were done in MATLAB with non linear least squares method and Levenberg-Marquardt algorithm.

The **Internal Weber Fraction** (***w***) was estimated fitting the dependence of error rates with log distance to the theoretical prediction given by equation 1 and 2. This was done collapsing across all SOA values for both tasks orders. Fits were done on a subject by subject basis to estimate *w* for each individual subject. The obtained individual values were then averaged (Figure 2A,D) and submitted to statistical analysis. Unless otherwise indicated, the **internal reference**–i.e. the numerical distance at which participants respond at chance–was set to the **objective reference** (fixed to 20 throughout the experiment). For certain fits (Figure 2B,E) the *log distance between the internal and the objective reference* simply referred as ***z*** was considered a free parameter (equation 2).

## Supporting Information

Appendix S1 Derivation of the equation to fit distribution of error rates
(0.09 MB DOC)
