# Supplementary material for: Delays without Mistakes: Response Time and Error Distributions in Dual-Task
Source: PLoS One. 2008 Sep 12;3(9):e3196. doi: 10.1371/journal.pone.0003196 (PMC2527526; doi:10.1371/journal.pone.0003196)
Supplement: Appendix S1 — Derivation of the equation to fit distribution of error rates (0.09 MB DOC) [file pone.0003196.s001.doc]

## APPENDIX S1

### Derivation of equation to fit distribution of error rates

To fit distribution of errors as function of evidence we derive the final equation from the expression for *P_larger_* derived in Dehaene (2007).


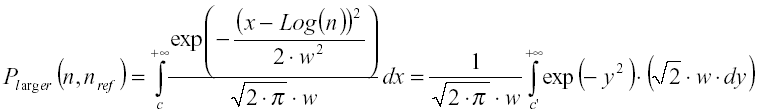
 (equation A1)

where,


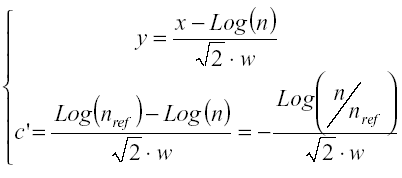
 (equation A2)

And we obtain:


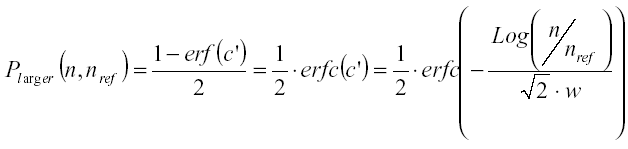
 (equation A3)

If the reference is not considered to be fixed then equation A1 needs to be modified to:


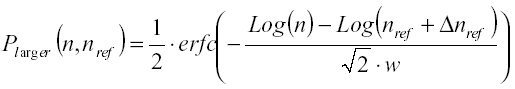
 (equation A4)


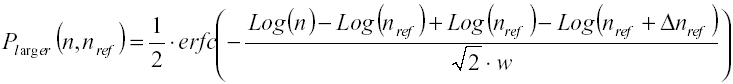
 (equation A5)


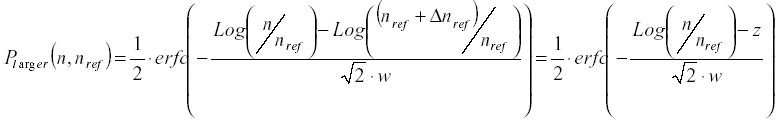
 (equation A6)

Where,


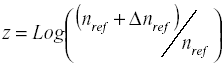
 (equation A7)

The internal representation (*n_ref_*  *+ Δn_ref_*) can be calculated:


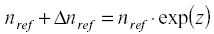
 (equation A8)

Finally, the probability to responded “larger” as function of Log distance (*r*) can be fitted as,


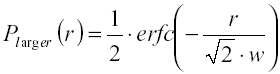
 (equation A9)


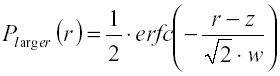
 (equation A10)

Note that equation A9 has a single free parameter, *w*, while equation A10 had two free parameters: *w* and *z*.
